# Genomic Characterization of HLJDZD55: The First L1B PRRSV in China

**DOI:** 10.1155/2024/2969771

**Published:** 2024-05-31

**Authors:** Jinhao Li, Hu Xu, Chao Li, Jing Zhao, Bangjun Gong, Qi Sun, Zhenyang Guo, Siyu Zhang, Menglin Zhang, Lirun Xiang, Yan-dong Tang, Jianan Wu, Qian Wang, Jinmei Peng, Guohui Zhou, Huairan Liu, Tongqing An, Xuehui Cai, Zhi-Jun Tian, Hongliang Zhang

**Affiliations:** State Key Laboratory for Animal Disease Control and Prevention Harbin Veterinary Research Institute Chinese Academy of Agricultural Sciences Harbin 150001China

## Abstract

Porcine reproductive and respiratory syndrome virus (PRRSV) critically threatens the pig industry in China. Lineage 1 PRRSV, which is divided into L1A–L1F and L1H–L1J, is widely recognized as the most extensively genetically diverse lineage globally. L1A (NADC34-like) and L1C (NADC30-like) PRRSVs have become the current major circulating strains in China. Notably, strains from other branches of L1 have not been reported in China. During our epidemiological investigation of PRRSV, we identified a new strain named HLJDZD55. Phylogenetic analysis of the ORF5 gene revealed that HLJDZD55 belongs to the L1B branch. Alignment of deduced amino acid sequences based on the Nsp2 gene indicated that HLJDZD55 has a discontinuous deletion of 131 amino acids (111 + 1 + 19). We further sequenced the whole genome of HLJDZD55, and phylogenetic analysis based on the whole-genome sequence revealed that HLJDZD55 belongs to the L1C branch. Recombination analysis of the whole genome demonstrated that HLJDZD55 is a recombinant strain of TJZH-1607 (L1C, identified in China) and Minnesota 14 (L1B, identified in the USA). These findings suggested that HLJDZD55 is a newly emerged lineage 1 PRRSV in China and is closely related to L1B PRRSV in the US, which may have been introduced from the U.S. strain and subsequently recombined with the local Chinese strain and underwent evolution. Taken together, these results demonstrated the emergence of L1B PRRSV in China for the first time.

## 1. Introduction

Porcine respiratory and reproductive syndrome (PRRS) is a major disease in the pig industry that causes considerable economic losses to the swine industry worldwide [1]. It is primarily characterized by reproductive failure in sows and respiratory diseases in piglets [2]. The causative agent of PRRS is the PRRS virus (PRRSV), which is widely prevalent worldwide and belongs to the genus *Betaarterivirus*, family *Arteriviridae*, and order *Nidovirales* [3]. PRRSV can be divided into two species, *Betaarterivirus suid 1* (PRRSV-1) and *Betaarterivirus suid 2* (PRRSV-2), which share 60% nucleotide (nt) identity at the whole-genome level [3]. Both PRRSVs are prevalent in China. Since the first isolation of PRRSV in 1996, PRRSV-2 has been predominant in China [4]. Rich genetic diversity is a crucial characteristic that develops during the spread of PRRSV [5].

PRRSV-2 was divided into lineages 1–11 based on the ORF5 gene [6]. Lineage 1 has become the most prevalent and diverse lineage within the American and Asian swine industries [7, 8]. Recently, based on an analysis of more than 82,000 global PRRSV-2 ORF5 sequences, American researchers have further refined the phylogenetic classification system and divided lineage 1 into nine sublineages (L1A–L1F and L1H–L1J) [6, 7]. Among lineage 1, L1A, L1B, and L1C are the most widely distributed and account for the largest proportion [6]. L1A and L1C are found in large numbers in North America, South America, and East Asia, while L1B is mainly detected in North America [6]. Sublineages of other branches are primarily distributed in the United States but can also be found in small quantities in other countries and regions, such as Mexico, Canada, Thailand, and South Korea [6].

From 1996 to 2006, the classic PRRSV strain (CH-1a, the representative strain, L8.7) was predominantly prevalent in China [4]. In 2006, highly pathogenic PRRSV (HP-PRRSV, L8.7) mutant strains emerged in China [9, 10]. Since 2013, a new PRRSV strain, named NADC30-like PRRSV, which is an L1C strain, has emerged in China [11, 12, 13]. This virus exhibits a discontinuous deletion of 131 amino acids in the Nsp2 protein [11, 12]. NADC30-like PRRSV is characterized by low homology, frequent recombination, and crucial variations in pathogenicity and has gradually become the predominant epidemic strain in China [14]. In 2017, another PRRSV strain, named NADC34-like PRRSV, which is an L1A strain, was first reported in China [15], which shares a continuous deletion of 100 amino acids in the Nsp2 protein [16]. Subsequently, the presence of such strains was also detected in southern China [17]. The coexistence of multiple genotypes of PRRSV will lead to occasional viral recombination [11, 15, 18]. For lineage 1, NADC30-like PRRSV has exhibited a significant tendency to recombine with strains from other genetic backgrounds [18, 19, 20] and has displayed rapid genomic divergence via recombination [21]. Along with the rapid spread of L1A and L1C PRRSV and the emergence of mutant strains resulting from recombination, these strains have gradually evolved into the major epidemic strains in China [22, 23, 24, 25], and there has been an increase in reports about them, but there are no reports on other sublineages of the L1 branch in China; the emergence of other branch strains also requires attention.

In this study, for the first time, we identified a new strain belonging to the L1B branch of PRRSV-2 through an epidemiological survey in China and conducted a comprehensive analysis of the genetic evolution and characteristics of the genome of this strain.

## 2. Materials and Methods

### 2.1. Source of Disease Materials and Sample Collection

In 2023, blood and lung samples were collected from approximately 20% of piglets with respiratory symptoms at a pig farm in Heilongjiang Province. Five samples (three serum samples and two lung samples) were collected from the piglets. Tissue sample collection and virus isolation were conducted following previously described methods [26, 27]. Cells used to isolate PRRSV include MARC-145, PAM, and PBMC cells. MARC-145 cells are a monkey kidney cell line that is highly susceptible and preserved by our laboratory. PAMs and PBMC were obtained from 4-week-old specific pathogen-free (SPF) pigs and cultured in RPMI 1640 medium (Gibco BRL Co., Ltd., USA) supplemented with 10% fetal bovine serum (FBS, ExCell Bio., Australia).

### 2.2. Genome Sequencing

Tissue sample processing, RNA extraction, cDNA preparation, RT-PCR, and genome sequencing were performed as previously described [26, 27]. The ORF5 gene in the samples was detected and amplified using specific primers (*Supplementary 1*). Amplification products from positive samples were sequenced and analyzed. To obtain the whole-genome sequence, eight pairs of full-length primers were used for amplification (*Supplementary 1*). The whole-genome sequence was assembled using MegAlign and Seqman.

### 2.3. Genomic and Phylogenetic Analysis

The genome and deduced amino acid sequences of the Nsp2 gene alignment were assessed using the ClustalW method in DNASTAR (version 7.1) software. Phylogenetic trees were generated, and molecular evolutionary analyses were conducted using the neighbor-joining method in MEGA 7.0 with 1,000 bootstrap replications [28].

### 2.4. Genomic Recombination Analysis

RDP4 and NCBI BLAST results were utilized to initially identify the major and minor parent strains [29]. The RDP4 software package incorporates seven algorithms (RDP, GeneConv, booscan, MaxChi, Chimera, SiScan, and 3Seq), which employ Bonferroni correction to detect recombination events and breakpoints [30]. To establish crucial evidence of recombination, at least four out of the seven detection methods for RDP4 were employed [30]. The identified recombination events were further validated using SimPlot 3.5.1 [29]. Boot scan analysis was conducted using a 200 bp window sliding along the genome sequence with a step size of 20 bp [29]. Phylogenetic trees were constructed based on different recombinant fragments to verify genetic evolution.

## 3. Results and Discussion

In 2023, we detected a novel PRRSV in Heilongjiang Province and conducted a detailed characterization of it. We collected three serum samples and two lung samples from piglets, where approximately 20% of the piglets exhibited respiratory symptoms. One serum sample and two lung samples were positive for PRRSV, and the Nsp2 and ORF5 genes were sequenced. Notably, the PRRSV sequences obtained from this farm were the same (data not shown), and we selected one of the strongly positive samples (from the lungs), HLJDZD55, for further study.

**Figure 1:**
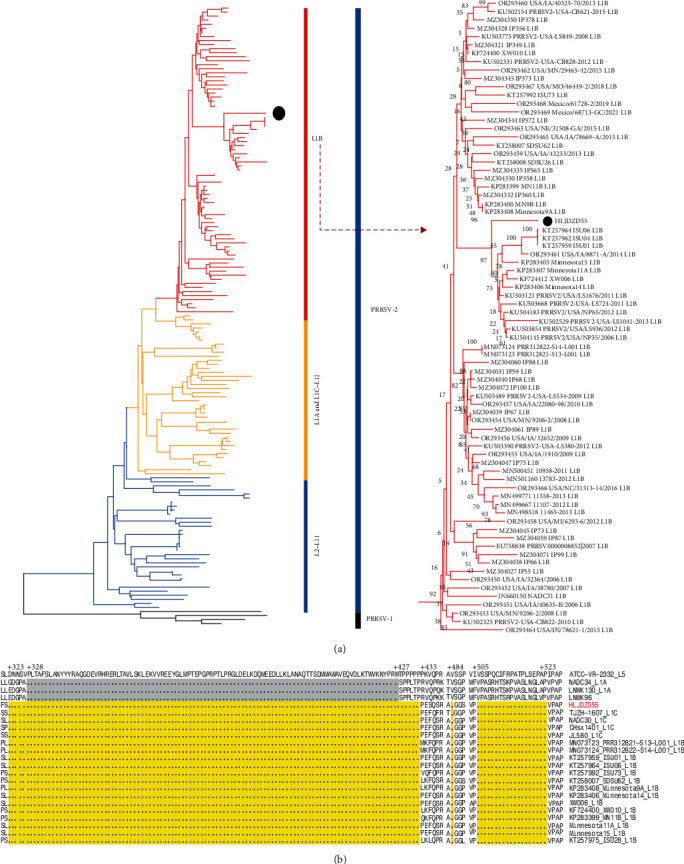
Genotyping of newly emerged PRRSV in China. (a) Phylogenetic tree constructed based on the ORF5 gene of HLJDZD55 and reference PRRSV strains from each lineage. The red branches represent the L1B strains, the yellow branches represent the L1A and L1C–L1J strains, and the blue branches represent the L2–L11 strains. HLJDZD55 was classified as belonging to the L1B branch and is labeled with ●. (b) Alignment of the deduced amino acid sequence based on the Nsp2 gene. The positions marked in the figure represent positions of the Nsp2 amino acid sequence and refer to the position of ATCC VR2332. Gray indicates the NADC34-like PRRSV 100 aa characteristic ontinuous deletion. Compared with the VR2332 Nsp2 gene, the HLJDZD55 and L1B strains have a discontinuous deletion of 131 amino acids (111 + 1 + 19), which are marked in yellow. HLJDZD55 is labeled in red.

Since the diversity of PRRSV-2 is usually measured by analyzing the variation in the open reading frame 5 (ORF5) gene [31, 32], which is a common method used in phylogenetic analysis [5], the Nsp2 gene exhibits the highest genetic diversity among PRRSV strains [33] and is used as a molecular marker to distinguish strains [34]. To investigate its classification, a phylogenetic tree was constructed using Chinese and global 152 PRRSV-1 and PRRSV-2 strains obtained from GenBank. Phylogenetic analysis of the ORF5 gene revealed that HLJDZD55 belongs to the L1B branch within L1 (Figure 1(a)). Deduced amino acid alignment based on the Nsp2 gene indicated a discontinuous deletion of 131 amino acids (111 + 1 + 19) in the Nsp2 gene of HLJDZD55, and this deletion is at the same position as that in the L1B and L1C PRRSVs (Figure 1(b)). The above analysis showed that this PRRSV strain differed from previously identified strains in China. According to available reports, the L1B strain has not been detected in China. Unfortunately, we were unable to successfully isolate the virus from PAM, MARC-145, and PBMC cells after repeated attempts. Moreover, a change in the cell tropism of the L1-branch PRRSV is a common phenomenon in vitro [26, 35, 36]. The reason or mechanism underlying this change needs further study.

**Figure 2:**
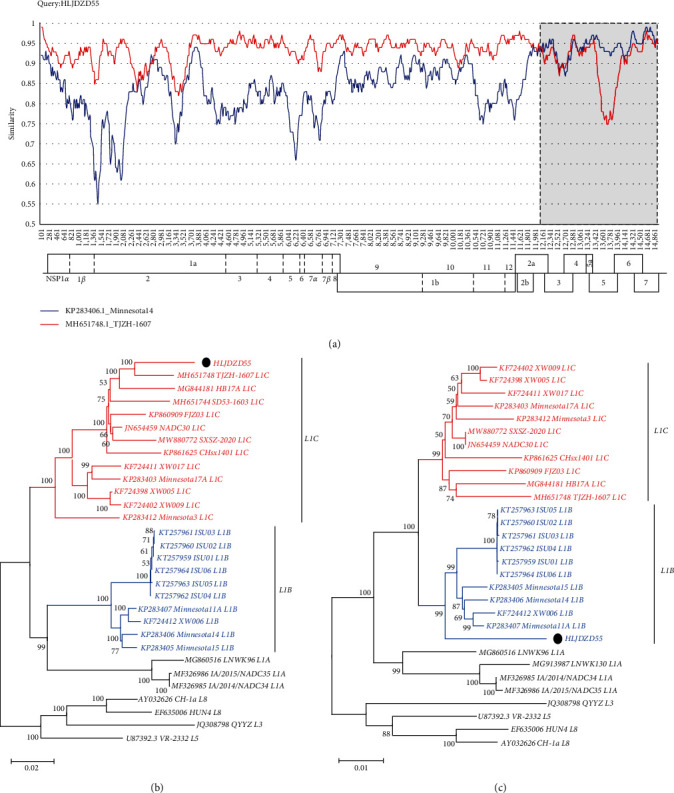
Recombination analysis of strain HLJDZD55. (a) Comparison of the similarity of HLJDZD55 and potential parental viruses using SimPlot. Recombination breakpoints are shown as black dotted lines. The background color of the major parental region (TJZH-1607) is white, whereas that of the minor parental region (Minnesota 14) is gray. TJZH-1607 and Minnesota 14 are shown in red and blue, respectively. The phylogenies of the major parents (b) and the minor parents (c) are shown as the similarity plots. The major parental groups (TJZH-1607 and L1C) are shown in red, while the minor parental groups (Minnesota 14 and L1B) are shown in blue; the other lineages are shown in black. HLJDZD55 is labeled with ●.

Whole-genome sequence analysis will provide a broader perspective on PRRSV evolution and expand the ability to identify recombinant PRRSV [6, 37]. To further analyze the genome characteristics of HLJDZD55, we designed eight primers to amplify and sequence its whole genome. Sequencing revealed that the length of the genome, excluding that of poly A, was 15012 nt. The phylogenetic analysis results based on the whole-genome gene sequences suggested that HLJDZD55 was classified as L1C (*Supplementary 2*); however, it belongs to L1B based on the ORF5 gene. Recombination is a pervasive phenomenon among PRRSV strains [38], so we preliminarily determined that HLJDZD55 may have undergone recombination and then performed a recombination analysis of the genome based on strains representative of each branch. RDP4 analysis revealed recombination events, and the HLJDZD55 strain was derived from L1C PRRSV and L1B PRRSV (*Supplementary 3*). BLAST analysis revealed that the nonstructural protein region of the HLJDZD55 sequence has high homology with that of the NADC30-like strain TJZH-1607 (MH651748, identified in China), and the structural protein region has high homology with that of the L1B strain Minnesota 14 (KP283406, identified in the USA); thus, it is most likely to have originated from these two strains. The recombination event was confirmed by SimPlot (version 3.5.1), which revealed a single recombination breakpoint in ORF2a (nt 12074) (Figure 2(a)). The putative recombination event was further supported by phylogenetic trees based on different recombinant fragments (Figures 2(b) and 2(c)). The homology analysis of the whole-genome sequences between HLJDZD55 and representative PRRSV strains indicated that HLJDZD55 shares the highest homology (93.4%) with the TJZH-1607 strain, and the homology with other representative strains is less than 90% (Table 1). Further analysis of each ORF gene sequence revealed that the ORF2-7 gene exhibited the highest homology with the Minnesota 14 gene (94.1%) (Table 1), which was consistent with the results of the recombination analysis of the genome. The above results indicated that HLJDZD55 may be a recombinant strain of Minnesota 14 and TJZH-1607 and that this strain has further evolved in China.

The L1B branch of PRRSV-2 emerged in North America in the early 2000s and primarily spread between the two midwestern regions of the United States [6, 39]. From 2002 to 2019, the proportion of L1B gradually increased, becoming dominant, and then finally decreased and stabilized [6]. Between 2009 and 2021, the L1B PRRSV became the dominant sublineage within the Mexican L1 PRRSV [6]. The L1C branch of PRRSV-2 is believed to have originated in Canada and spread from Canada to the upper midwest in the early 21st century, after which it gradually expanded to other regions within the United States [39, 40]. After a certain degree of spread and evolution, it mostly existed in North America and East Asia and became a dominant sublineage in these regions [6, 39]. L1C (NADC30-like) PRRSV appeared in China in 2013 and caused large losses [11, 13]. The virus rapidly spread to many regions of China with high detection rates [15, 24, 41]. Due to the extensive genetic diversity and complex recombination of NADC30-like PRRSV, the pathogenicity of NADC30-like strains varies significantly [24, 42, 43]. HLJDZD55 (Chinese L1B branch PRRSV) is a recombinant strain of Minnesota 14 (America L1B branch PRRSV) and TJZH-1607 (Chinese L1C branch PRRSV). The origin of this strain may be that the American L1B branch PRRSV was introduced in China and then further recombined with the Chinese L1C branch PRRSV. The discovery of new strains reminds people who that it is very important for China to strengthen the monitoring, prevention and control of L1B PRRSV and newly imported strains.

## 4. Conclusion

In this study, for the first time, we report a novel PRRSV belonging to the L1B branch that emerged in China. Its structural proteins are most closely related to L1B PRRSV, and its nonstructural proteins are most closely related to Chinese NADC30-like PRRSV. We speculate that Chinese L1B PRRSV was introduced from American L1B PRRSV, subsequently recombined with Chinese L1C PRRSV, and further underwent evolution.

## Figures and Tables

**Table 1:** Comparison of ORFs and proteins of the HLJDZD55 and the representative strains of other lineages or sublineages.

HLJDZD55	TJZH-1607	Minnesota 14	CH-1a	NADC34	QYYZ	VR-2332	HUN4
Complete genome	93.4	86.7	83.4	84.7	81.4	84.4	82.6
5′UTR	98.4	90.3	90.5	93.6	87.3	91.0	89.4
ORF1a	93.1	82.9	80.0	81.0	77.6	81.5	78.8
ORF1b	94.7	87.2	86.7	87.2	84.4	86.6	85.7
ORF2-7	91.8	94.1	86.1	88.8	85.0	87.1	86.2
3′UTR	97.3	95.2	87.2	93.9	87.8	91.9	89.2
Nsp1*α*	92.4	88.3	86.9	88.5	86.9	88.3	85.9
Nsp1*β*	92.6	81.4	78.1	81.8	74.3	80.2	78.4
Nsp2	92.0	82.6	74.1	77.1	73.0	77.8	75.1
Nsp3	95.9	81.9	83.0	86.2	80.7	84.6	81.4
Nsp4	95.3	84.2	85.0	82.4	83.7	85.6	84.5
Nsp5	93.1	80.2	89.6	83.1	80.4	87.1	87.5
Nsp6	93.8	87.5	91.7	85.4	91.7	89.6	93.8
Nsp7*α*	93.1	80.8	81.9	81.9	79.4	85.9	81.0
Nsp7*β*	94.2	83.6	79.1	81.8	77.9	80.6	77.9
Nsp8	97.1	89.1	87.7	90.6	84.8	88.4	88.4
Nsp9	94.9	88.3	86.0	88.2	84.1	87.1	85.5
Nsp10	94.0	89.3	85.4	89.5	84.2	85.5	84.4
Nsp11	93.7	82.4	90.9	82.8	85.1	87.1	88.5
Nsp12	97.0	83.3	87.2	82.7	85.3	87.2	86.4
ORF2a	94.3	93.4	86.1	84.3	86.3	87.4	85.9
ORF2b	96.4	91.4	87.8	87.4	89.6	88.7	88.3
ORF3	92.7	92.8	82.9	86.0	82.6	82.5	82.9
ORF4	94.8	95.0	87.9	95.3	85.7	88.6	87.7
ORF5	83.4	94.7	85.4	88.2	80.9	85.6	85.7
ORF5a	92.0	97.1	88.4	91.3	84.8	87.7	87.0
ORF6	92.8	94.5	87.0	90.7	89.3	89.9	88.4
ORF7	96.0	97.8	90.9	95.4	87.6	92.5	90.9

## Data Availability

The sequence of this study was deposited in GenBank with the accession number PP395769. The sequence will be released to public databases when the data or accession number appear in print. The sequence data are supplied in the supplementary files.

## Supplementary Materials

Supplementary 1 Primers used for detection and amplification of the full-length genome of HLJDZD55 PRRSV.

Supplementary 2 Phylogenetic tree constructed based on the full-length genome of HLJDZD55 and reference PRRSV strains of other lineages. The red branches represent strains of the L1C branch, and the black branches represent strains of other branches. HLJDZD55 was classified as belonging to the L1C branch and is labeled with ●.

Supplementary 3 Information on recombination events of HLJDZD55 PRRSV detected by RPD4 software.
