# Supplementary material for: Genomic Characterization of HLJDZD55: The First L1B PRRSV in China
Source: Transbound Emerg Dis. 2024 May 31;2024:2969771. doi: 10.1155/2024/2969771 (PMC12020383; doi:10.1155/2024/2969771)
Supplement: Supplementary 1 — Primers used for detection and amplification of the full-length genome of HLJDZD55 PRRSV. [file 2969771.f1.docx]

Table S1 Primers used for detection and amplification of the full-length genome of HLJDZD55 PRRSV

| Fragment | Primer sequence (5’-3’) | Position in genome | Product size(bp) |
| --- | --- | --- | --- |
| PRRSV-ORF5 ^a^ | ATGTTGGGGAAATGCTTGACCG  CTAAGGACGTCCCCATTGTTC | 13,390-13,992 | 603 |
| PRRSV-Nsp2 ^a^ | GGTGCTGGGAAGAGAGCAAGAA  GCCTGTTCTCCGGAAGGCTCT | 1,337-2,360 | 1,024 |
| PRRSV-I ^b^ | ATGACGTATAGGTGTTGGCTC  CAACTCCAGGAAGCACAACA | 1-2,225 | 2,225 |
| PRRSV-II ^b^ | AGTGTGTCCAGGGTTGCTGT  GAAACACGTTAAAGGCGACCT | 1,758-4,034 | 2,277 |
| PRRSV-III ^b^ | AGTTTGACTCGCCAGAGTGT  CATTCCGAAGGAGAAAACACT | 3,753-5,983 | 2,230 |
| PRRSV-IV ^b^ | AGTCACATAATTAGAGACGTA  AGACCCCTAGCGATACTGGGAG | 5,756-7,820 | 2,064 |
| PRRSV-V^b^ | AGACCCCTAGCGATACTGGGAG  GATTGCAATACGCCGCTTCGT | 7,799-9,913 | 2,114 |
| PRRSV -VI ^b^ | AGGTCCAGGATGGTGATGTCA  CGGCAGAGCGCGCACGGAGT | 9,704-11,869 | 2,165 |
| PRRSV-VII ^b^ | AGGCTCGAACTGAAATGAAAT  GTACCCGGCGGTAGACACAGT | 11,661-13,692 | 2,031 |
| PRRSV-Ⅷ ^b^ | ATTCTGTTTTGCTGCGCTCGT  TTAATTACGGCCGCATGGTTCT | 13,455-15,012 | 1,557 |

^a^ Primers used for detection of HLJDZD55 PRRSV; ^b^ Primers used for amplification the whole genome of HLJDZD55 PRRSV. The primer sequences, the position in genome and product size were based on TJZH-1607 (MH651748) strain.
