# Supplementary material for: Genomic Characterization of HLJDZD55: The First L1B PRRSV in China
Source: Transbound Emerg Dis. 2024 May 31;2024:2969771. doi: 10.1155/2024/2969771 (PMC12020383; doi:10.1155/2024/2969771)
Supplement: Supplementary 2 — Phylogenetic tree constructed based on the full-length genome of HLJDZD55 and reference PRRSV strains of other lineages. The red branches represent strains of the L1C branch, and the black branches represent strains of other branches. HLJDZD55 was classified as belonging to the L1C branch and is labeled with ●. [file 2969771.f2.docx]

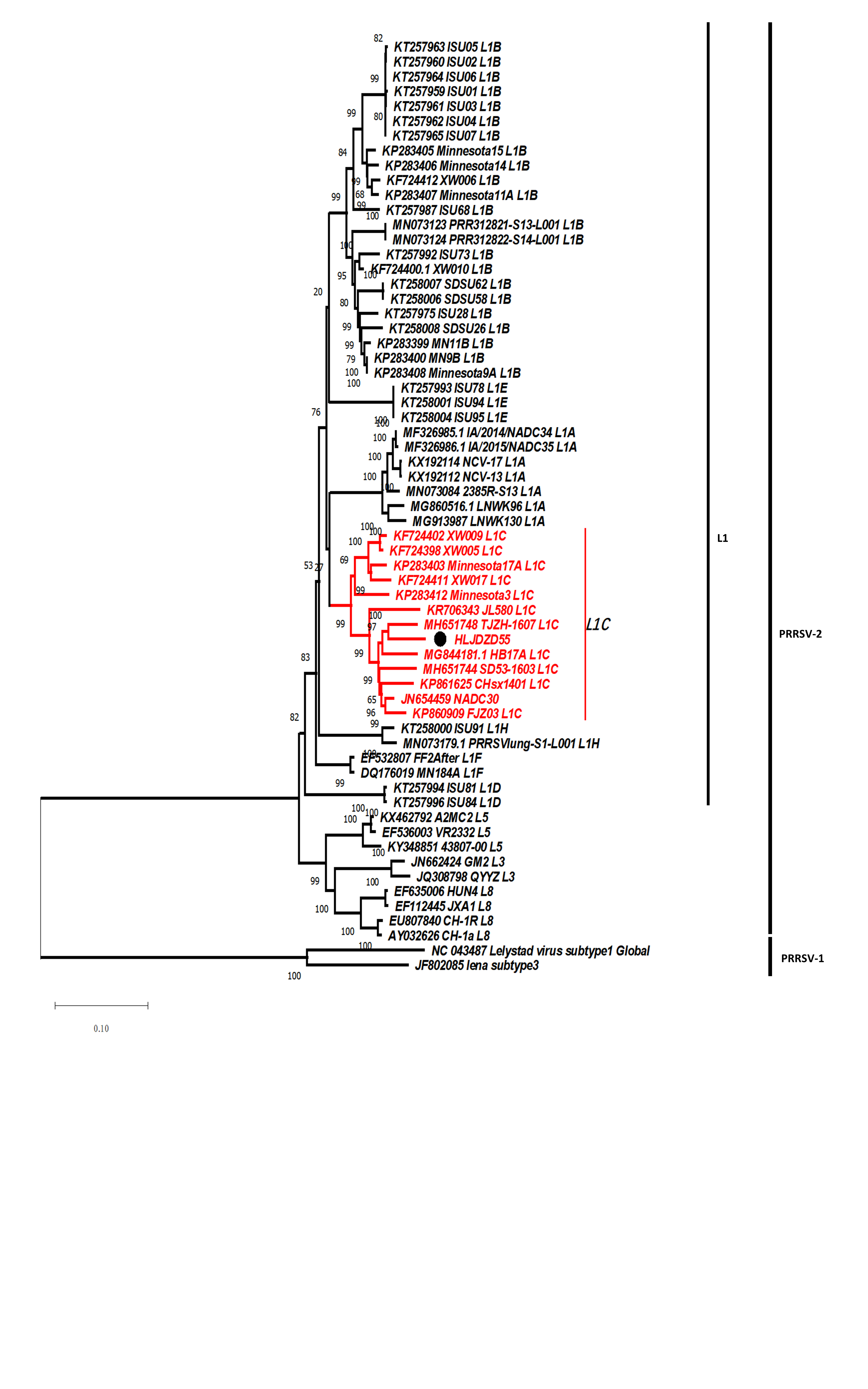


Fig. S1. Phylogenetic tree constructed based on the full-length genome of HLJDZD55 and reference PRRSV strains of other lineages. The red branches represent strains of the L1C branch, and the black branches represent strains of other branches. HLJDZD55 was classified as belonging to the L1C branch and is labeled with ●.
