# Supplementary material for: Genomic Characterization of HLJDZD55: The First L1B PRRSV in China
Source: Transbound Emerg Dis. 2024 May 31;2024:2969771. doi: 10.1155/2024/2969771 (PMC12020383; doi:10.1155/2024/2969771)
Supplement: Supplementary 3 — Information on recombination events of HLJDZD55 PRRSV detected by RPD4 software. [file 2969771.f3.docx]

Table S2 Information on recombination events of HLJDZD55 PRRSV detected by RPD4 software.

| Strains | Breakpoints | | Parental Sequence | | Detection Methods (p-V alue) | | | | | | |
| --- | --- | --- | --- | --- | --- | --- | --- | --- | --- | --- | --- |
|  | Beginning | Ending | Minor | Major | RDP | GENECONV | BootScan | MaxChi | Chimaera | SiScan | 3Seq |
| HLJDZD55 | 12074 | 15012 | Minnesota 14 | TJZH-1607 | 1.481×10^-33^ | 2.842×10^-12^ | 1.863×10^-19^ | 1.335×10^-30^ | 9.508×10^-4^ | - | 1.559×10^-10^ |

-: not significant
